# Anxiolytic Efficacy of Indirubin: *In Vivo* Approach Along with Receptor Binding Profiling and Molecular Interaction with GABAergic Pathways

**DOI:** 10.1002/open.202400290

**Published:** 2024-10-25

**Authors:** Ishrat Jahan Disha, Rubel Hasan, Shimul Bhuia, Siddique Akber Ansari, Irfan Aamer Ansari, Muhammad Torequl Islam

**Affiliations:** ^1^ Biochemistry and Molecular Biology Bangabandhu Sheikh Mujibur Rahman Science and Technology University Gopalganj 8100 Bangladesh; ^2^ Bioinformatics and Drug Innovation Laboratory BioLuster Research Center Ltd. Gopalganj, Dhaka 8100 Bangladesh; ^3^ Department of Pharmacy Bangabandhu Sheikh Mujibur Rahman Science and Technology University Gopalganj 8100 Bangladesh; ^4^ Department of Pharmaceutical Chemistry College of Pharmacy King Saud University Riyadh 11451 Saudi Arabia; ^5^ Department of Drug Science and Technology University of Turin Turin 10124 Italy; ^6^ Pharmacy Discipline Khulna University Khulna 9208 Bangladesh

**Keywords:** Anxiety, Indirubin, Diazepam, Molecular docking, *in vivo* study

## Abstract

Anxiety is a natural response to stress, characterized by feelings of worry, fear, or unease. The current research was conducted to investigate the anxiolytic effect of indirubin (IND) in different behavioral paradigms in *Swiss* albino mice. To observe the animal's behavioural response to assess anxiolytic activity, different tests were performed, such as the open‐field (square cross, grooming, and rearing), swing, dark‐light, and hole cross tests. The experimental mice were administered IND (5 and 10 mg/kg, p.o.), where diazepam (DZP) and vehicle were used as positive and negative controls, respectively. In addition, a combination treatment (DZP+IND‐10) was provided to the animals to determine the modulatory effect of IND on DZP. Molecular docking approach was also conducted to determine the binding energy of IND with the GABA_A_ receptor (α2 and α3 subunits) and pharmacokinetics were also estimated. The findings revealed that IND dose‐dependently significantly (*p*<0.05) reduced the animal's movement exerting calming behavior like DZP. IND also demonstrated the highest docking score (−7.7 kcal/mol) against the α3 subunit, while DZP showed a lower docking value (−6.4 kcal/mol) than IND. The ADMET analysis revealed that IND has proper drug‐likeness and pharmacokinetic characteristics. In conclusion, IND exerted anxiolytic effects through GABAergic Pathways.

## Graphical abstract

Anxiolytic activity of indirubin was tested on mice through the open‐field test (the number of square crosses, grooming, and rearing), swing test, dark residence time (DRT), and number of hole crosses (NHC). The findings indicated that indirubin demonstrated calming effects and higher docking score (−7.7 kcal/mol) against the α3 subunit manifesting anxiolytic like effects.

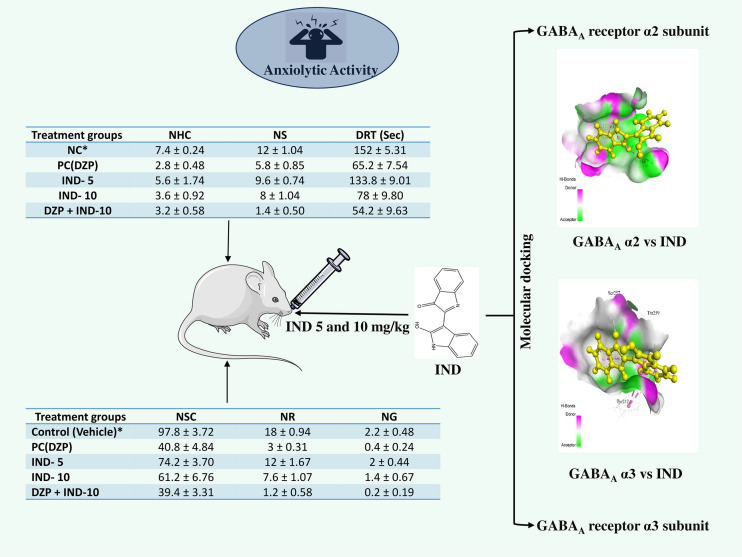

## 1 Introduction

Neurodegenerative disorders are defined by the gradual deterioration of the structure and function of the nervous system, specifically the neurones of the brain.^[87]^ These disorders often include the progressive degeneration of neurones and their interconnections, resulting in a deterioration of cognitive abilities, motor control, or both^[3]^. In addition, anxiety disorders include a range of psychological issues that are often associated with symptoms of depression.[^54^, ^82^] Though their emotional characteristics are similar, anxiety and depression vary in a number of ways. Approximately 4 % of the world's population is now suffering from an anxiety condition. In 2019, anxiety disorders accounted for 301 million cases globally, making them the most prevalent kind of mental illness (https://www.who.int/news‐room/fact‐sheets/detail/anxiety‐disorders, accessed on 15 April, 2024).

However, anxiety has a substantial risk factor for multiple diseases and disorders, such as neuropsychiatric, cardiovascular, and metabolic problems.^[25]^ It can arise from numerous factors, including mental conditions, stressful lifestyles, physical status, alcohol abuse, genetics, and the harmful impact of drugs. (Bhuia et al., 2023).^[24]^

Several neurotransmitters may be involved in the pathophysiological mechanisms of anxiety disorders, such as dysregulation or abnormalities of norepinephrine (NE), serotonin (5HT), histaminergic, acetylcholine, glutamate, dopamine, and gamma‐aminobutyric acid (GABA), which are major factors.[^7^, ^36^, ^47^] GABA is the primary neurotransmitter responsible for inhibiting neural activity in the brain and is recognized for its role in regulating and balancing the transmission of excitatory signals in different neuronal systems.^[56]^ The regulation of anxiety‐related responses is significantly influenced by GABAergic neurotransmission in the amygdala.^[61]^ However, the anticonvulsant, amnesia, and sedative actions of benzodiazepines (BZDs) are caused by the activation of GABA type A (GABA_A_) receptors that include the α1‐subunit. On the other hand, the anxiolytic effects of BZD are linked to certain types of GABA_A_ receptors, including those that have the α2‐ and α3‐subunits,[^7^, ^49^, ^73^, ^74^] According to a study, anxiety behaviours may be regulated by α5‐containing GABA_A_ receptors expressed in some neuronal populations in the central amygdala.^[11]^ Consequently, this finding was transformed into anxiolytic‐like effects that were facilitated by systemic α5 modulations.^[5]^ The majority of studies have indicated that BZD‐induced anxiolysis is associated with the α2 subtype rather than the α3 subtype.^[16]^

Medications that affect the central nervous system (CNS), including BZDs and barbiturates such as diazepam, alprazolam, and lorazepam, are used for a variety of purposes, including anxiolytic, sedative, hypnotic, anticonvulsant, and muscle relaxant.^[40]^ BZD is widely administered worldwide due to its notable features and effectiveness. Consequently, they are also among the medicines that are often abused. The concurrent use of BZD with alcohol poses an increased risk due to its potential to substantially enhance sleepiness.^[60]^ In addition, selective serotonin reuptake inhibitors (SSRIs), serotonin‐norepinephrine reuptake inhibitors (SNRIs), and monoamine oxidase inhibitors (MAOIs) are also used to treat patients with neurological disorders. However, there are several concerns about their safety and tolerability.[^27^, ^31^] Although BZDs are highly efficacious in alleviating acute anxiety, they are associated with detrimental side effects such as aggression, unsteadiness, confusion, drowsiness, dizziness, irritability, and memory impairment.[^14^, ^58^] In this situation, alternative drugs that are derived from naturally occurring bioactive substances may be a promising remedy for anxiety and anxiety disorders (Bhuia et al., 2023).^[41]^ Chemicals extracted from plants, such as alkaloids, terpenes, flavonoids, phenolic acids, lignans, cinnamates, and saponins, have been shown to exhibit anxiolytic effects in different animal models.^[29]^

Indirubin (IND) (3‐(3‐oxo‐1H‐indol‐2‐ylidene)‐1H‐indol‐2‐one) is a key bioactive compound present in indigo plants. It is a purple 3,2′‐bis‐indole alkaloid that is obtained from Radix and *Folium itindidis*, which are widely grown in China, as well as from mollusks, human urine, and other microorganisms. It has been shown to possess several biological properties, including antitumor,^[92]^ anticancer,[^17^, ^30^] antiviral,^[59]^ hepatoprotective,^[90]^ antiallergic,^[48]^ stem cell regulator,^[20]^ antibacterial (Da et al., 2016), antifungal,^[64]^ neuroprotective,^[88]^ anti‐inflammatory agent for psoriasis,^[79]^ endotoxemia,^[67]^ antiparasitic activity,^[28]^ and antidiabetic activities.^[50]^ Additionally, it may have potential applications in treating neurodegenerative disease.^[57]^ The main objective of this study is to determine the anxiolytic effect of IND in *Swiss* albino mice. Simultaneously, a molecular docking study is performed to investigate protein‐ligand interactions that may be liable for the observed effects.

## 2 Materials & Methods

### 2.1 *In Vivo* (Animal) Study

#### 2.1.1 Chemicals and Reagents

IND (CAS No. 479–41‐4.) was collected from Sigma‐Aldrich (St. Louis, MO, USA), where the standard drug diazepam (DZP) obtained from Square Pharmaceuticals Ltd. and tween 80 required for this study were obtained from Merck (India).

#### 2.1.2 Preparation of Test Sample and Standards

Based on a literature review, we selected two concentrations of the test sample.^[93]^ We formulated the mother solution for the test sample at a concentration of 10 mg/kg by dissolving it into water for injection (WFI), where tween 80 was used as a co‐solvent. The mother solution was then diluted at a dose of 5 mg/kg. Additionally, the solution of standard drug (DZP) was prepared by vigorous mixing into WFI at dose of 2 mg/kg.

#### 2.1.3 Experimental Animals

In this investigation, we purchased the male *Swiss* albino mice (22–30 g) from the animal house at Jahangirnagar University in Bangladesh. These animals were placed in the pharmacology lab of Bangabandhu Sheikh Mujibur Rahman Science and Technology University (BSMRSTU), Gopalganj, 8100. They were openly accessed to receive the conventional granules as a base diet and water *ad libitum*. They were maintained under regulated lighting conditions (12‐hour light/dark cycle) at a temperature of 27±2 °C until the experiment began. The current test was conducted from 8:00 a.m. to 3:00 p.m., and the animals were monitored for 17 hours to determine their potential mortality following the research. The Department of Pharmacy of the BSMRSTU (#bsmrstu16‐11/22) granted approval for the experimental design and methods.

#### 2.1.4 Study Design

Experimental animals were required to remain fasting for at least six hours prior to the test. Then, in total, 25 animals were arbitrarily categorized into 5 groups, each containing 5 animals. Group‐I was served with a vehicle as the negative control (NC), and group‐II was administered with DZP‐2 mg/kg. On the other hand, groups III and IV were administered with IND‐5 and IND‐10 mg/kg, respectively. In addition, group‐V was considered for the combination treatment (DZP+IND‐10). The doses of the test sample and control treatments varied according to the weight of each animal. The various treatment groups and their respective doses are shown in Table 1.

**Table 1:** Different treatment groups and their perspective doses.

Treatment Group	Description (R/A) (p.o.)	Dose (mg/kg)
Gr‐I: NC	Distilled water containing 0.9 % NaCl and 0.5 % tween 80	10
Gr‐II: DZP‐2	Standard: Diazepam (agonist)	2
Gr‐III: IND‐5	Test sample: Indirubin (lower dose)	5
Gr‐IV: IND‐10	Test sample: Indirubin (upper dose)	10
Gr‐V: DZP+IND‐10	Test+Standard combination	2+10

NC: Negative Control; DZP: Diazepam (Dose: 2 mg/kg); IND: Indirubin (Dose: 5 and 10 mg/kg).

### 2.2 Anxiolytic Activity Study

#### 2.2.1 Open‐Field Test (OFT)

The open field apparatus was made of a wooden open field space with a pointed square surface (30×30×30 cm^3^). In a 5 minute period, we manually collected the number of square crossings (NSC), number of grooming (NG), and number of rearing (NR). Once the parameters for each animal were recorded, the experimental equipment was cleaned using 70 % ethanol.^[52]^

#### 2.2.2 Swing Test (ST)

The research was carried out using the methodology established and provided by Islam et al.^[42]^ Following the administration of the experimental treatments, each animal was positioned in the swing equipment. The frequency of swings produced by the natural motions of each animal inside the device was recorded. After each test, the surface of the equipment was sanitized using a solution of 65 % ethyl alcohol.

#### 2.2.3 Hole‐Cross Test (HCT)

In this study, we utilized a 30×20×14 cm^3^ wooden apparatus that contained a 3 cm diameter opening only one inch over the bottom of the cage's partition board. Following a 3 minute ST, each mouse was immediately positioned at one end of the hole‐board tool. For five minutes, mice were observed freely traversing the rooms through the opening. The mice passing through the hole were manually noted. As previously stated, the apparatus surface was meticulously cleaned.^[22]^

#### 2.2.4 Light‐Dark Test (LDT)

The testing equipment was constructed from wood and separated into two chambers: the light room and the dark room. A narrow door connected these rooms. The ambient light illuminates the lightbox (27×18×29 cm^3^), which is brighter compared to the dark box (black portion: 271,829 cm^3^). Right after a 3 minute HCT, every mouse was positioned at one end of the light‐dark apparatus. The duration of time (in seconds) that each animal spent in both dark and light conditions was measured for a period of 5 minutes using a stopwatch. After every test, the floor of the equipment was meticulously cleaned.^[34]^

#### 2.2.5 Statistical Analysis

The values are represented as the mean±standrd error of mean (SEM). The statistical analysis conducted was a one‐way ANOVA, followed by a t‐students Newman‐Keuls post‐hoc test. Multiple comparisons were made at a 95 % confidence level. The analysis was performed using GraphPad Prism software (version: 9.5, San Diego, USA). Significance was attributed to data with a p‐value of p<0.05.

### 2.3 *In Silico* Studies

#### 2.3.1 GABA Macromolecule Selection and Preparation

The Protein Data Bank (PDB) is a unique international institution that has gathered and provided open access to the 3D models of protein macromolecules and their complexes. This is an advanced online resource that focuses on innovative studies in the field of biology.^[12]^ The three‐dimensional conformations of GABA_A_ (α2 and α3) receptor subunits were obtained from the RCSB Protein Data Bank (https://www.rcsb.org/), accessed on April 10, 2024. The Φ‐Ψ Ramachandran plot was used in accordance with PROCHECK to evaluate the stereochemical characteristics of particular protein chains.^[83]^ In addition, the PDBsum database was used to authenticate the quality and authenticity of the receptors.^[26]^

#### 2.3.2 Ligand Preparation

**Figure 1:**
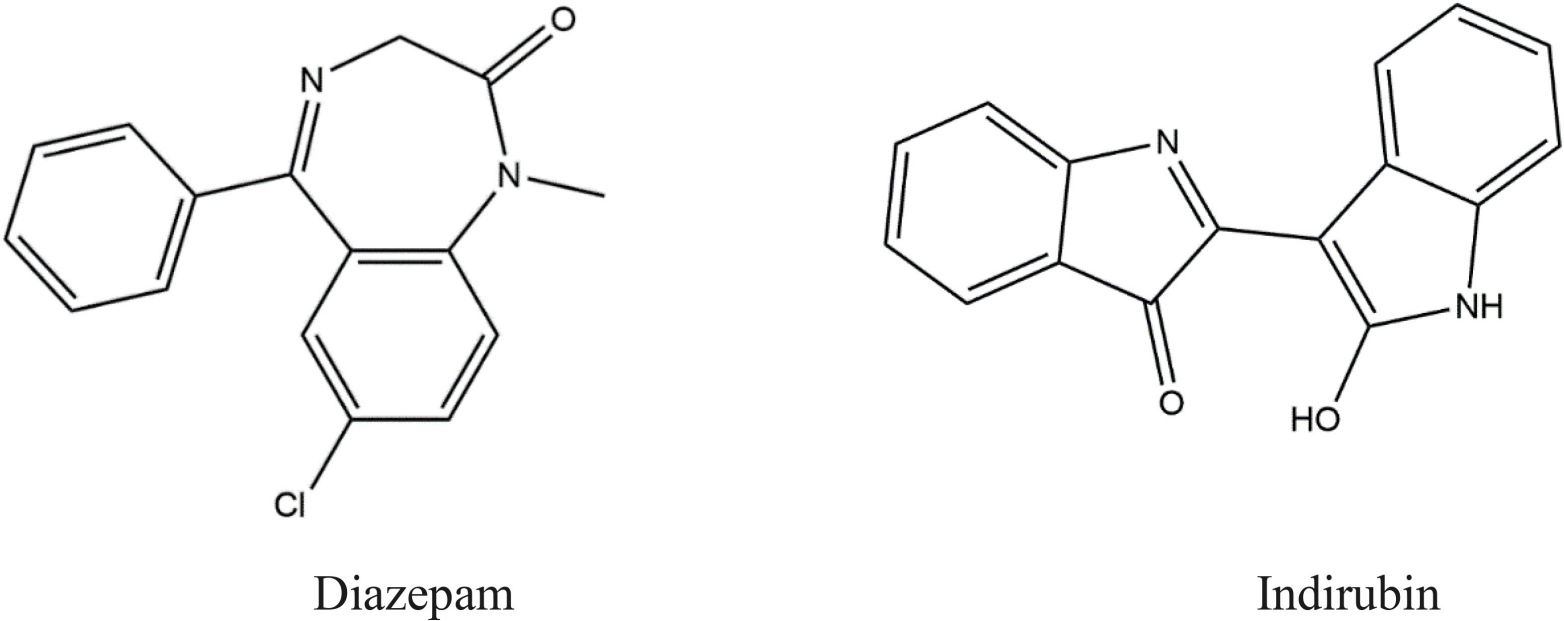
The 2D structures of diazepam and indirubin.

The 3D chemical structures of the conventional medication DZP (PubChem ID: 3016) and the compound IND (PubChem ID: 10177) were acquired from the PubChem database in sdf format. We used the Chem3D Pro20.1.1 program to perform ligand minimization utilizing Allinger's force field (MM2) approach.^[4]^ Figure 1 represents the 2D structures of diazepam and indirubin.

#### 2.3.3 Docking Protocol and Non‐Bond Interactions

Molecular docking study is a prevalent computer technique used in pharmaceutical research to facilitate the development of drugs.^[23]^ This approach evaluates the pharmacodynamic properties of medications by examining and establishing connections between molecules and specific binding locations using the PyRx v0.8 program.^[1]^ The result of the docking procedure reflects the intensity of the contact between a ligand and the active region of a certain protein. To expedite the docking approach, the dimensions of the grid box's X, Y, and Z axes were modified to 35.02×39.37×25.00 Å.[^19^, ^46^] The calculation was then performed 2000 times.^[51]^ The PDB format of the ligand‐protein complex was obtained to convert the ligand into PDBQT format.^[38]^ The previous research indicated that the binding affinity of the ligand was quantified as a negative value in kcal/mol.^[91]^ Furthermore, the protein's active binding regions are determined using BIOVIA Discovery Studio v21.1.0. This program enables the assessment of non‐bonded interactions in the complexes formed by ligands and proteins.^[8]^

#### 2.3.4 Pharmacokinetics and Drug‐Likeness Properties

Pharmacokinetics, an investigation of how a pharmacological component breaks down in the body, is often evaluated by analyzing the many factors that affect its ability to reach the intended target as separate parameters.^[44]^ Estimating the ADME features early in the discovery phase greatly reduces the incidence of pharmacokinetics‐related failures in the later clinical stages.[^65^, ^75^] The SwissADME web server is an effective instrument for estimating the ADMET parameters and drug‐likeness features of compounds.^[37]^

#### 2.3.5 Toxicity Prediction

Toxicity prediction is a crucial step in the drug discovery procedure that helps identify and prioritize compounds with the most promise for safe and effective use in humans. This strategy also aids in reducing the probability of costly failures in the subsequent phases of drug development.[^63^, ^86^] The ProTox 3.0 online server may be used to forecast different toxicity parameters for a particular medicine. To determine the toxicity properties, the canonical SMILES acquired from PubChem were sent to the ProTox 3.0 website (http://tox.charite.de/protox‐3). The server subsequently identified the toxicity classification and many properties of the chemical. The toxicity properties of selected chemicals are analyzed and described in Table 7.

## 3 Results

### 3.1*. In Vivo* Findings

#### 3.1 Open Field Tests

The findings of this study stated that the animals in the vehicle group (NC group) had the highest NSC (97.80±3.72), NR (18.00±0.94), and NG (2.20±0.48). On the other hand, the reference drug (DZP) significantly (*p* <0.05) diminished the value of NSC (40.80±4.84), NR (3.00±0.31), and NG (0.40±0.24) compared to the vehicle group. Additionally, the test sample (IND) demonstrated a dose‐dependent response on experimental animals. The test sample also significantly (*p*<0.05) reduced the mean value of different test parameters such as NSC (74.20±3.70), NR (12.00±1.67), and NG (2.00±0.44) for IND‐5 and NSC (61.20±6.76), NR (7.60±1.07), and NG (1.40±0.67) for IND‐10, respectively. Furthermore, the combination treatment (DZP+IND‐10) lowered all the test parameter values of NSC (39.40±3.31), NR (1.20±0.58), and NG (0.20±0.19) when compared to the DZP administered alone. The mean value of all the test samples is documented in Table 2.

**Table 2:** Number of square crosses, rearing, and grooming for control and other test groups.

Treatment groups	NSC	NR	NG
Negative control (Vehicle)*	97.8±3.72	18.00±0.94	2.20±0.48
PC(DZP)	40.8±4.84	3.00±0.31	0.40±0.24
IND‐5	74.2±3.70	12.00±1.67	2.00±0.44
IND‐10	61.2±6.76	7.60±1.07	1.40±0.67
DZP+IND‐10	39.4±3.31	1.20±0.58	0.20±0.19

PC: Positive Control; DZP: Diazepam; IND: Indirubin; NSC: number of square crosses; NR: number of rearing; NG: number of grooming.

#### 3.2 Hole Cross Test

The results of the hole cross test showed that the animals in the vehicle group had the maximum number of hole crosses (NHC) (7.40±0.24). Conversely, the NHC significantly (*p*<0.05) reduced (2.80±0.48) in the referral drug (DZP) treated animal's group compared to the vehicle group. Results also exhibited that the test sample belonging to the group significantly (*p*<0.05) declined the NHC in a dose‐dependent manner. The values of NHC are 5.60±1.74 and 3.60±0.92 for the IND‐5 and IND‐10, respectively. However, the combination therapies (DZP+IND‐10) displayed the NHC values (3.20±0.58) higher than the DZP administered alone. The mean value of the NHC for all the treatment groups is presented in Table 3.

**Table 3:** Number of hole‐crosses, swings, and time spent in dark chamber for control and other test groups.

Treatment groups	NHC	NS	DRT (Sec)
NC*	7.40±0.24	12.00±1.04	152.00±5.31
PC(DZP)	2.80±0.48	5.80±0.85	65.20±7.54
IND‐5	5.60±1.74	9.60±0.74	133.80±9.01
IND‐10	3.60±0.92	8.00±1.04	78.00±9.80
DZP+IND‐10	3.20±0.58	1.40±0.50	54.20±9.63

PC: Positive Control; DZP: Diazepam; NHC: number of hole‐cross; NS: number of swings; DRT: dark residence time.

#### 3.3 Swing Test

In this experiment, the vehicle group displayed the highest number of swings (NS) (12.00±1.04). On the other hand, the standard drug (DZP) significantly (*p*<0.05) decreased the NS (5.80±0.85) compared to the vehicle group. In addition, our result demonstrated a dose‐dependent response in the animals treated with the test sample (IND). The mean values of NS for the groups of IND‐5 and IND‐10 are 9.60±0.74 and 8.00±1.04, respectively. Moreover, the animals who received a combined treatment (DZP+IND‐10) showed the lowest NS value (1.40±0.50) compared to the entire treatment group. The mean value of the NS for all groups is documented in Table 3.

#### 3.4 Dark‐Light Study

Our findings in the dark resident time (DRT) study showed that the vehicle group animals remained the maximum time (152.00±5.31 sec) in the dark box, while the DZP treated animals’ group significantly (p<0.05) diminished in DRT (65.20±7.54 sec) compared to the vehicle group. Additionally, the lower doses of the test sample (IND‐5) demonstrated the DRT value of 133.80±9.01 sec, while at the same time, higher doses of the test sample (IND‐10) significantly (*p* <0.05) reduced the DRT (78.00±9.80 sec) compared to the vehicle group. However, the combination therapies (DZP+IND‐10) exhibited the lowest DRT (54.20±9.63 sec) among all the treatment groups. The mean value of DRT for various treatment groups is documented in Table 3.

### 3.1 In Silico Studies

#### 3.1.1 Macromolecules of GABA_A_ Receptor Subunits GABA_A_ Receptor Homology Modelling

**Figure 2:**
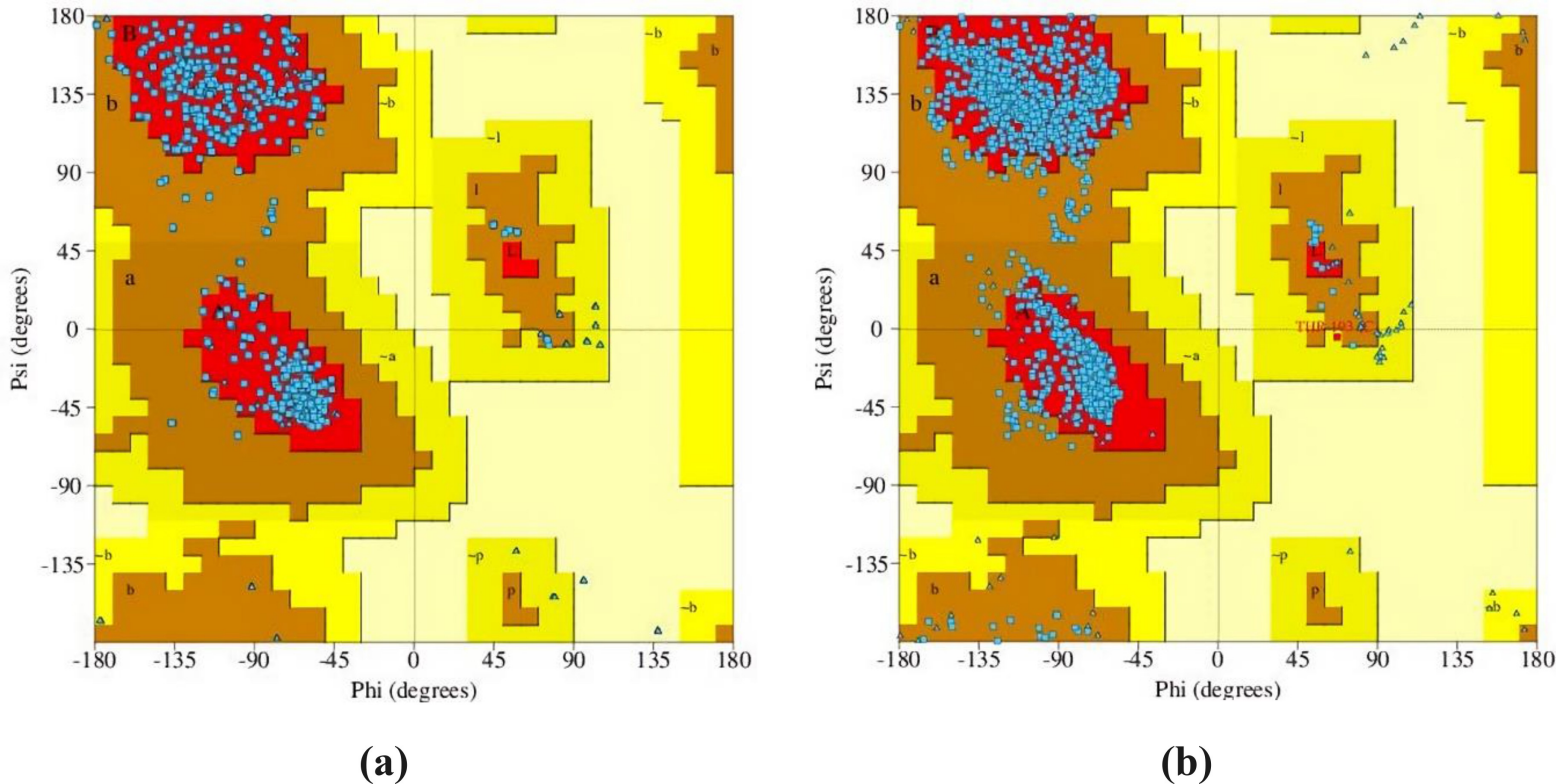
Ramachandran plot of the homology modelled GABA_A_ receptor (a) α2 subunit; (b) α3 subunit.

The homology modelling findings indicate that the target sequences of the GABA_A_ receptor α2 and α3 subunits exhibit a similarity of 57.18 % and 76.50 %, respectively, when compared to the template sequence. The homology model of the human GABA_A_ receptor was developed with GMQE values of 0.60 and 0.66 for the α2 and α3 subunits, respectively. This indicates that the developed receptors are of high quality and can be relied upon. The precision and uniformity of the residues’ Psi and Phi angles were evaluated using the Ramachandran plot. The GABA_A_ receptor α2 and α3 subunits have extra approved sites of 7.30 % and 8.0 %, respectively. The figure shows that the most desired areas of the modelled receptors make up 92.70 % and 91.90 % of the display. The banned regions of both receptor subunits were expressed at a rate of 0.00 %0 % in the plot (Figure 2).

#### 3.1.2 Molecular Docking and Visualization of Ligand‐Receptor Interactions

##### 3.1.2.1 Interaction of Indirubin with GABAA Receptor Subunits

**Figure 3:**
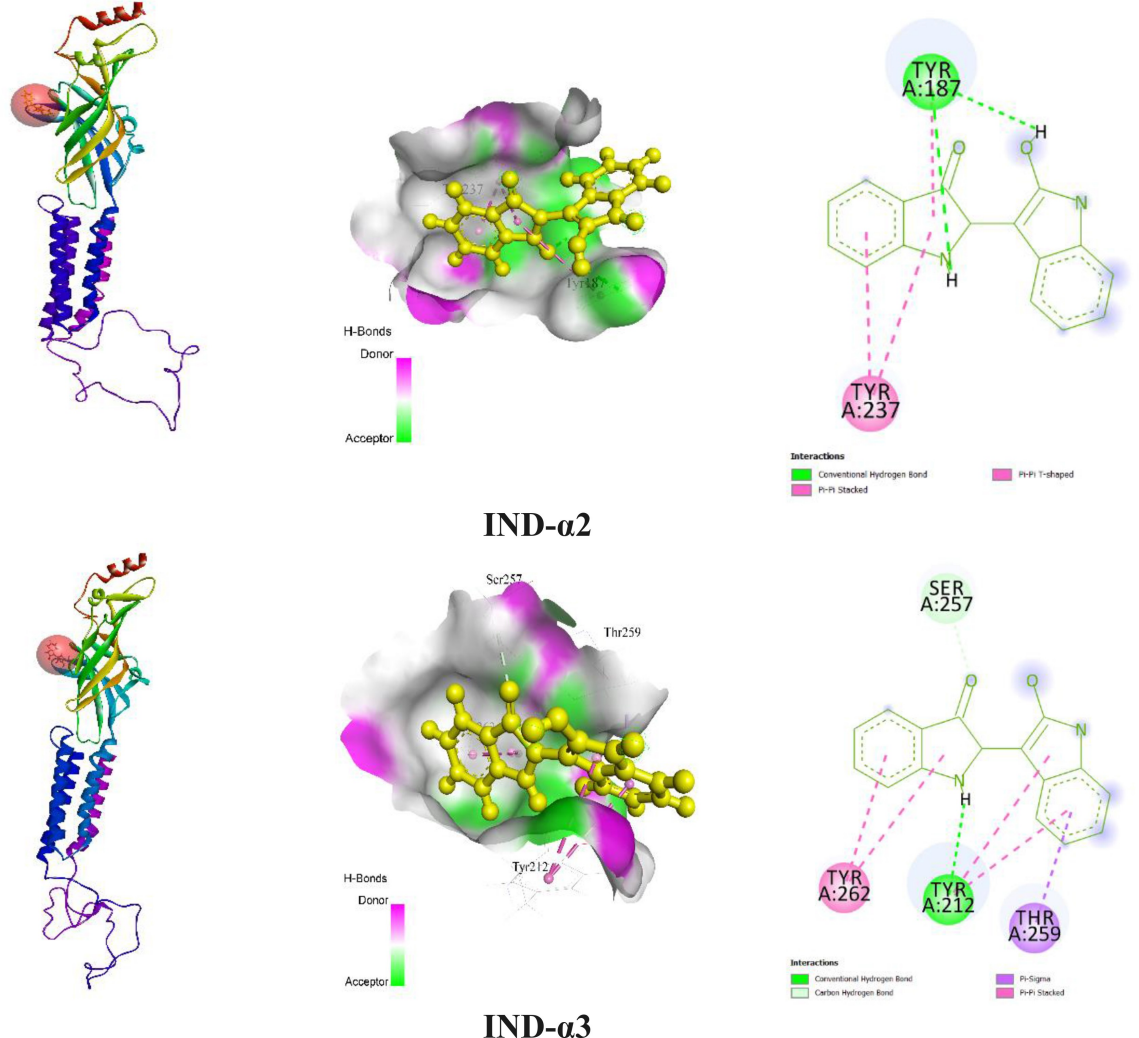
The 3D and 2D views of protein‐ligand interaction and their binding sites with the associated amino acid residues. [IND: Indirubin].

Table 4 showed the findings of our *in silico* study that during the interaction of the tested ligand (IND) with the GABA_A_ receptor (α2 and α3) subunits, they formed several types of chemical bonds, including hydrogen bonds (HB), hydrophobic (HP) bonds, and other bonds. IND demonstrated the highest docking score (−7.7 kcal/mol) against the GABA_A_ receptor (α3) subunit through two HBs (TYR212 and SER257), one Pi‐Sigma (THR259), and two Pi‐Pi Stacked (TYR212 and TYR262). IND also revealed a higher binding energy (−7.1 kcal/mol) towards the GABA_A_ receptor (α2) subunit. In addition, IND formed one HB (TYR187), one Pi‐Pi Stacked (TYR237) and one Pi‐Pi T‐shaped (TYR187). Docking score and HB lengths are documented in Table 4 and the 2D and 3D structures of IND non‐bond interactions with GABA_A_ α2 and GABA_A_ α3 are depicted in Figure 3.

**Table 4:** Evaluating the docking and identifying the amino acid residues involved in the interaction between ligands and receptors.

Ligand	Receptors	Docking Scores (Kcal/mol)	No of HB	Amino acid Residues	
				HB	Others
IND	GABA_A_ α2	−7.1	1	TYR187	TYR237 (Pi‐Pi Stacked), TYR187 (Pi‐Pi T‐shaped)
	GABA_A_ α3	−7.7	2	TYR212, SER257	THR259 (Pi‐Sigma), TYR212 (Pi‐Pi Stacked), TYR262 (Pi‐Pi Stacked)

IND: Indirubin; HB: Hydrogen bond.

##### 3.1.2.2 Interaction of Diazepam with GABAA Receptor Subunits

**Figure 4:**
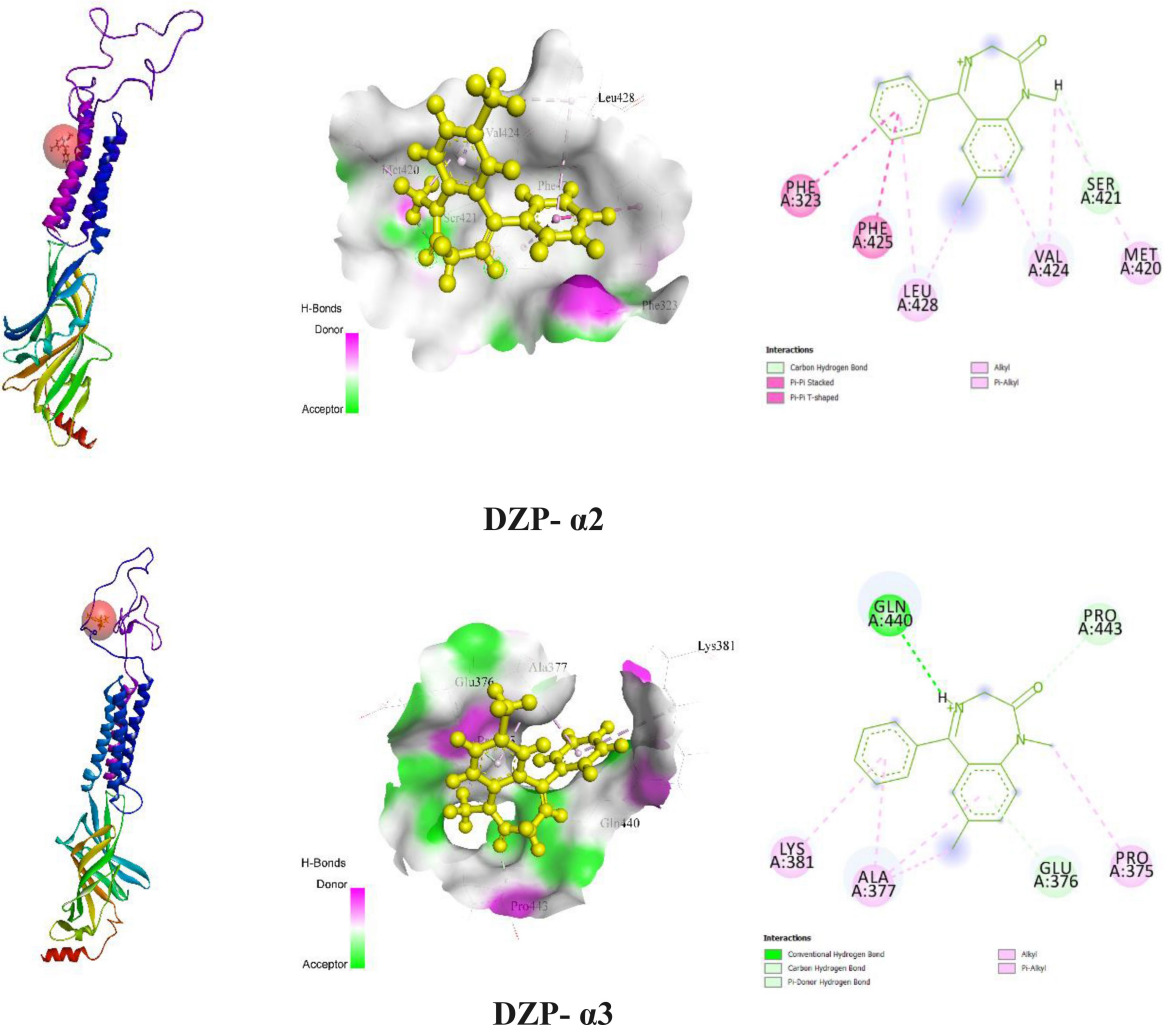
The 3D and 2D views of protein–ligand interaction and their binding sites with the associated amino acid residues. [DZP: Diazepam].

The *in silico* study displayed a number of HBs, HP bonds, and other bonds by expressing specific amino acid (AA) residues when standard ligands (DZP) connect with the GABA_A_ receptor (α2 and α3) subunits. DZP exhibited a higher binding value (−7 kcal/mol) towards the α2 subunit better than the α3 subunit of the GABA_A_ receptor. Additionally, DZP connects with the GABA_A_ receptor of the α2 subunit via one HB (SER421), one Pi‐Pi‐stacked (PHE425), one Pi‐Pi‐T‐shaped (PHE323), three alkyls (LEU428, MET420, and VAL424), and two Pi‐Alkyl (LEU428 and VAL424). Moreover, DZP required a binding energy of −6.4 kcal/mol to bind with the GABA_A_ receptor of the α3 subunit. Moreover, DZP interacts with the α3 subunit by forming three HBs (GLN440, PRO443, and GLU376), two alkyls (ALA377 and PRO375), and two Pi‐Alkyl (ALA377 and LYS381). Table 5 documented the molecular docking result and their AA residues; Figure 4 illustrated the 2D and 3D configuration of DZP and GABA_A_ receptors.

**Table 5:** Evaluating the docking and identifying the amino acid residues involved in the interaction between ligands and receptors.

Ligands	Receptors	Docking Scores (kcal/mol)	No of HB	Amino acid Residues	
				HB	Others
DZP	GABA_A_ α2	−7	1	SER421	PHE425 (Pi‐Pi Stacked), PHE323 (Pi‐Pi T‐shaped), LEU428 (Alkyl), MET420 (Alkyl), VAL424 (Alkyl), LEU428 (Pi‐Alkyl), VAL424 (Pi‐Alkyl)
	GABA_A_ α3	−6.4	3	GLN440, PRO443, GLU376	ALA377 (Alkyl), PRO375 (Alkyl), ALA377 (Pi‐Alkyl), LYS381 (Pi‐Alkyl)

DZP: Diazepam; HB: Hydrogen bond.

#### 3.1.3 Pharmacokinetic and Drug‐Likeness Characteristics (SwissADME)

**Figure 5:**
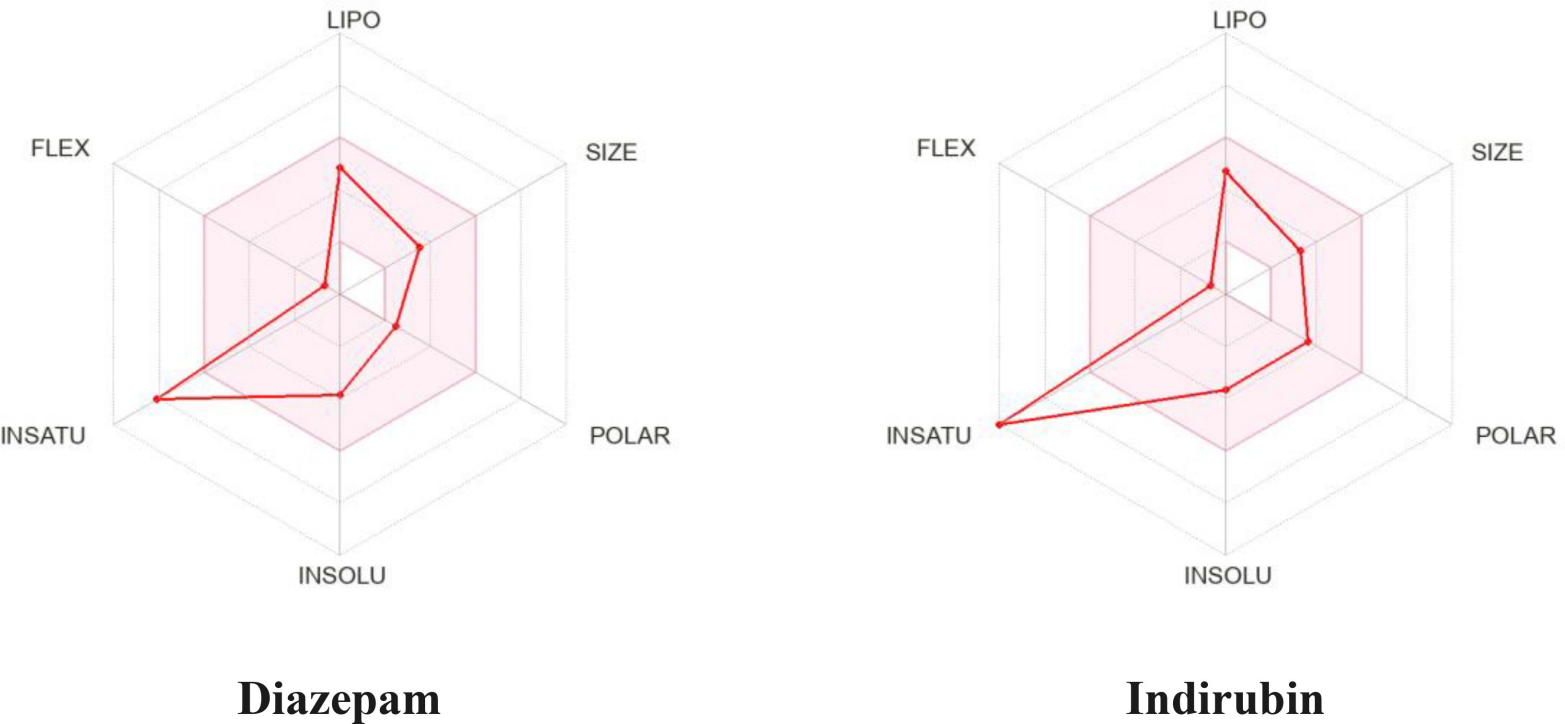
Summary of physiochemical properties of diazepam and indirubin. [The colored zone is the suitable physicochemical space for oral bioavailability; SIZE: 150 g/mol<MV<500 g/mol; INSOLU (Insolubility): −6<log S (ESOL) <0; LIPO (Lipophilicity): −7<XLOGP3 <+5.0; INSATU (Insaturation): 0.25<Fraction Csp^3^<1; POLAR (Polarity): 20 Å^2^<TPSA<130 Å^2^; FLEX (Flexibility): 0<num. rotatable bonds<9].

The *in silico* SwissADME investigation showed that the selected compound (IND) and referral drug (DZP) both have the molecular weight (MW) below 500 Dalton. In addition, DZP and IND contain the number of hydrogen bond acceptors (HBA) (2 and 3 respectively). This study also displayed that IND only consists of 2 hydrogen bond donors (HBD), while DZP does not have any HBD. Thus, our findings predicted that both chemicals accomplished all the criteria of Lipinski's rule. Results also demonstrated that both compounds had the ability to penetrate the blood‐brain barrier (BBB). At the same time, they exhibited high absorption properties in the gastrointestinal (GI) tract. Moreover, DZP and IND are soluble in water. Furthermore, DZP and IND show robust drug‐likeness attributes, adhering to essential criteria like those established by Ghose, Veber, Egan, and Muegge, which suggests their promise as potential drug candidates. Apart from this other parameter, such as TPSA, P‐gp substrate, Log P*o/w* (XLOGP3), Log P*o/w* (MLOGP), bioavailability score, Fraction Csp3, and molar refractivity of DZP and IND, are included in Table 6 and the graphical illustration shown in Figure 5.

**Table 6:** The physicochemical and pharmacokinetic characteristics of diazepam and indirubin predicted by SwissADME.

Parameters	DZP	IND
Physicochemical properties		
MF	C_16_H_13_ClN_2_O	C_16_H_10_N_2_O_2_
MW	284.74 g/mol	262.26 g/mol
Number of heavy atoms	20	20
Number of aromatic heavy atoms	12	15
Fraction Csp^3^	0.12	0.00
HBA	2	3
HBD	0	2
TPSA (Å^2^)	32.67 Å^2^	65.45 Å^2^
MR	87.95	80.62

Solubility		
Solubility (water)	Soluble	Soluble
Lipophilicity		
Log P*o/w* (XLOGP3)	2.99	2.73
Log P*o/w* (MLOGP)	2.67	1.70

Pharmacokinetics		
GI absorption	High	High
BBB permeant	Yes	Yes
P‐gp substrate	No	No
BIO Score	0.55	0.55
CYP2C19 int	Yes	No

Medicinal chemistry		
Synthetic accessibility	3.00	2.84
Drug likeness		
Lipinski	Yes; 0 violation	Yes; 0 violation
Ghose	Yes	Yes
Veber	Yes	Yes
Egan	Yes	Yes
Muegge	Yes	Yes

MF: Molecular formula; LogP: Log P*o/w* (MLOGP) (optimum: ≤5); MW: Molecular weight (g/mol) (optimum: ≤500); HBA: Hydrogen bond acceptor (optimum: ≤10); MR: Molar refractivity (optimum: ≤140); HBD: Hydrogen bond donor (optimum:≤5); CYP2 C19 int: CYP2 C19 inhibitor; TPSA: Topological polar surface area; BIO Score: Bioavailability Score.

#### 3.1.4 Toxicological Profile (ProTox 3.0)

Based on the *in silico* toxicity evaluation, DZP is categorised as toxicity class 2, while IND falls into toxicity class 4. The LD_50_ value of DZP is substantially lower at 48 mg/kg, in contrast to IND's LD_50_ value of 1500 mg/kg. Our results also exhibited that the standard drug (DZP) and our selected chemical (IND) were shown to have negative outcomes (inactive) in the cases of immunotoxicity, mutagenicity, and cardiotoxicity. In addition, DZP was inactive in the context of hepatotoxicity, carcinogenicity, and nephrotoxicity, while IND exhibited a positive result (active). However, IND didn't show the toxic property in terms of cytotoxicity, respiratory toxicity, and ecotoxicity, whereas DZP showed a positive outcome (active). The toxicity characteristics and corresponding values for the used chemical substances are provided in Table 7.

**Table 7:** Estimation of different toxicity parameters of DZP and IND using ProTox 3.0 online server.

Properties	Parameters	DZP	IND
Toxicity	LD_50_	48 mg/kg	1500 mg/kg
	Toxicity class	2	4
	Hepatotoxicity	Inactive	Active
	Carcinogenicity	Inactive	Active
	Immunotoxicity	Inactive	Inactive
	Mutagenicity	Inactive	Inactive
	Cytotoxicity	Active	Inactive
	Neurotoxicity	Active	Active
	Nephrotoxicity	Inactive	Active
	Respiratory toxicity	Active	Inactive
	Cardiotoxicity	Inactive	Inactive
	Ecotoxicity	Active	Inactive

DZP: Diazepam; IND: Indirubin.

## 4 Discussion

Anxiety is a prevalent emotional state marked by feelings of concern, apprehension, or frustration, usually related to an impending occurrence or something with an unpredictable result. Additionally, if anxiety reaches an extreme or persistent level, it may disrupt everyday functioning and perhaps signify the presence of an anxiety disorder.[^13^, ^72^] However, the assessment of anxiety‐like actions in mice has mostly been conducted using a limited number of traditional animal models of anxiety, such as the light/dark choice or the open‐field tests. These approaches rely on exposing individuals to new and unpleasant environments.^[6]^

The open field test (OFT) is a widely used method for evaluating exploratory behaviour and physical activity levels in mice and rats. The open field (OF) is primarily an enclosed area, often square, rectangular, or circular in form, with walls that impede escape. The primary and prevalent result of interest is “movement or locomotion”.[^18^, ^35^, ^78^] A current study indicated that rats with lower levels of locomotor activity may exhibit an anxiolytic status.^[81]^ In the OFT, anxiolytic drugs diminish the inclination of animals to engage in new situations by decreasing their locomotor activity.^[66]^ BZD enhances the effects of the neurotransmitter GABA in the brain, leading to a decrease in the movement of animals during testing and promoting a calming effect.^[77]^ Our *in vivo* investigation demonstrated that the animals belonging to the both drugs DZP and IND significantly diminished the various parameters such as NSC, NR, and NG in the OFT; this indicates a decrease in locomotor activity, resulting in reduced movement and calming behaviours. The results also demonstrated that mice receiving the IND in all groups exhibited dose‐dependent acquisition of relaxing behaviours.

To assess the CNS activities of animals, the swing protocol can be used; it offers the locomotor activities. A reduction in the animal's locomotion inside the swing apparatus indicates a calming, depressing, or sedative effect.^[42]^ On the other hand, the hole cross test is a commonly used experimental approach for studying emotional responses, namely the anxiolytic effects. Moving animals often pass through the opening in the enclosed container.[^43^, ^70^] The animals that show a significant reduction in their movements and a reduced desire regarding the new surroundings are considered to be calm. The significant decrease in spontaneous motion might be interpreted as an anxiolytic effect due to the stimulation of the GABAergic system.[^43^, ^68^] According to our study, the tested compound (IND) group and the standard (DZP) group decreased swing box movement and hole crossings compared to the vehicle group, indicating a calming effect. Our results indicate that animals treated with IND have a response that varies depending on the dosage.

The light/dark box (often known as the black and white box) is commonly used in mice to assess anxiety levels. The test equipment has two chambers of the same size that are joined by a tunnel.^[62]^ In this case, mice are put in the light chamber and given the freedom to move between the two compartments, and the time they spend in the dark chamber is recorded as the DRT.^[80]^ When reduced, the DRT indicates the animals exhibited a preference for familiar surroundings, namely the light chamber. This shows that the test compounds had a calming effect, leading to anxiolytic properties.^[85]^ Our experiment revealed that the vehicle group animals spent more time in the dark compartment; conversely, IND and DZP‐treated animals remarkably reduced the residence time in the dark compartment, indicating animals preferred to stay in a familiar environment (the light chamber) and lowered their movement. In this case, the test sample showed a dose‐dependent response. A very recent study showed that IND protected retinal glioblastoma cells from oxidative stress and mitigated retinal neurodegeneration due to organic neuronal cell injury. It also exerted its neuroprotective effects mostly via the PI3 K/AKT/BAD/BCL‐2 signalling pathway.^[57]^

**Figure 6:**
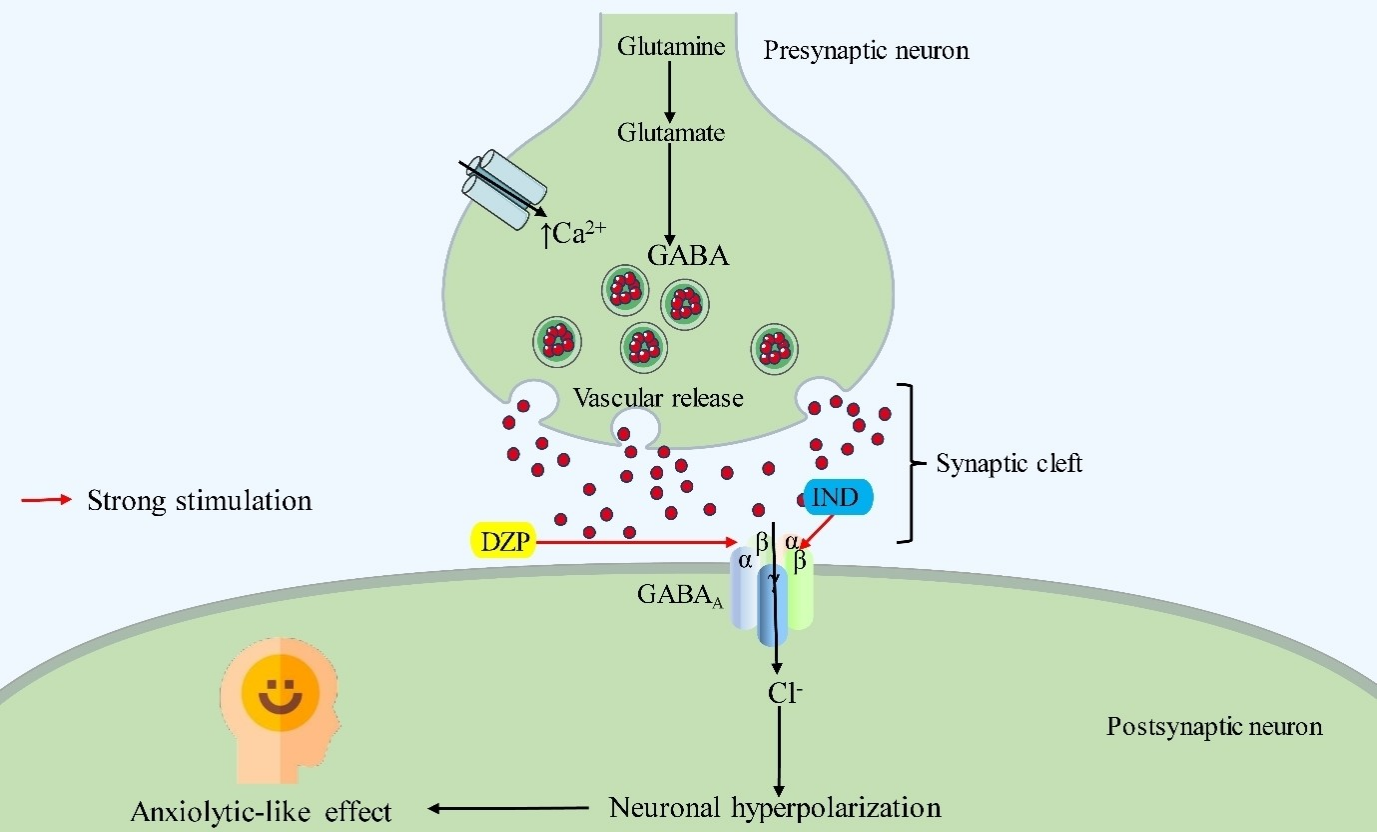
The suggested anxiolytic mechanism of the test compound (IND) compared to the selected standard drugs [DZP: Diazepam; GABA: Gamma aminobutyric acid; IND: Indirubin; This figure illustrates the possible anxiolytic mechanisms of IND and DZP based on their binding affinity with the α2 and α3 subunits of the GABA_A_ receptor. Here, DZP and IND act as GABA receptor agonists. Dysregulation of GABA levels is associated with several neurological and mental health conditions, including anxiety. If GABA levels are increased, there will be no anxiety].

A synergistic effect refers to a phenomenon where the combined action or cooperation of multiple elements or substances produces a result greater than the sum of their individual effects.^[89]^ Our results from this investigation exhibited that the combination therapies (DZP+IND‐10) diminished the value of NSC, NR, and NG but alleviated the NHC compared to the DZP treated alone. Additionally, our result also showed that the value of NS and DRT was diminished compared to the DZP. From the entire results of the combination treatments in several tests, it was determined that the locomotor activity of the test animals was reduced in all circumstances in the combined group, except the NHC test. Thus, our data indicates that the IND had a synergistic impact on experimental animals. Figure 6 depicts the potential molecular anxiolytic mechanism of IND. A proposed mechanism of action if IND is illustrated in Figure 6.

Computer‐aided drug design (CADD) is a contemporary computer method used in the process of drug development to find and develop a possible lead compound. CADD encompasses the use of computational chemistry, molecular modelling, molecular design, and rational drug development. It is now being used to enhance the efficiency of discovered lead compounds.[^39^, ^76^] It also reduces expenses related to animals and laboratories, as well as the overall duration of the examination. Molecular affinity is a measure used to evaluate the strength of the binding interaction between a ligand and a certain protein.^[32]^ Our computer‐based investigation demonstrated that DZP and IND specifically interacted with the α2 and α3 subunits of the GABA_A_ receptor. The result of this molecular docking study is that IND interacts with the α3 subunit of the GABA_A_ receptor with the highest docking value (−7.7 kcal/mol) and also forms two HBs with the specific AA residues (TYR212 and SER257). In addition, the IND also creates three HP bonds by expressing the available AA resides of THR259 (Pi‐Sigma), TYR212 (Pi‐Pi Stacked), and TYR262 (Pi‐Pi Stacked). On the other hand, standard ligand (DZP) showed a lower docking score (−6.4 kcal/mol) against the α3 subunit of the GABA_A_ receptor. During the receptor ligand interaction, they create three HBs with certain AA residues (GLN440, PRO443, and GLU376). Furthermore, the results also revealed that the IND displayed a higher binding score (−7.1 kcal/mol) towards the GABA_A_ receptor (α2 subunit). The IND interacted with the α2 subunit of the GABA_A_ receptor through a single HB and three HP bonds by expressing specific AA residues of TYR187 and TYR237 (Pi‐Pi Stacked) and TYR187 (Pi‐Pi T‐shaped), respectively. Conversely, the DZP demonstrated the docking score (−7 kcal/mol) due to the binding with the GABA_A_ receptor α2 subunit. Moreover, they form one HB (SER421). However, the higher the docking value of a chemical towards a particular receptor, that indicates a better interaction with that specific receptor.^[84]^ Therefore, our *in silico* study suggests that IND exerts anxiolytic‐like effects by interacting with the α3 subunit of the GABA_A_ receptor pathway.

The preclinical process that is necessary to create and utilize the molecules of interest as medication refers to computational drug design. Several computational methods, such as pkCSM and SwissADME, enable evaluating the ADMET characteristics of a substance.[^2^, ^53^] An ideal drug candidate must possess both potent effectiveness contrary to the therapeutic target and proper ADMET characteristics at a therapeutic dosage.^[33]^ The Lipinski's rule of five is widely used for the prediction of pharmacokinetics and drug‐likeness. Lipinski's rule of five states that a drug candidate should have a molecular weight (MW) of 500 g/mol or less, no more than five HBD, no more than ten HBA, and a lipophilicity (LogPo/w) of five or less.^[55]^ According to our *in silico* ADMET analysis, both ligands are projected to possess exceptional pharmacokinetic properties and fall within the range of becoming potential drugs according to Lipinski's criteria. The selected ligand fulfils all the criteria of Lipinski's rule of five and demonstrates an excellent pharmacokinetic property.

Chemical compound toxicity is predicted through the utilization of computational models in *in silico* toxicity analysis.^[69]^ Without requiring significant laboratory testing, this method can yield useful insights into the possible adverse effects of substances. In order to predict the acceptability of small molecules for application in animal and human models, it is essential to conduct a toxicological assessment.[^10^, ^15^] According to our *in silico* toxicity prediction, the selected compound didn't show any adverse effect in terms of mutagenicity, immunotoxicity, cytotoxicity, respiratory toxicity, cardiotoxicity, or ecotoxicity. Additionally, the projected LD_50_ value of IND (1500 mg/kg) was greater than DZP (48 mg/kg), indicating that IND is less harmful than DZP when provided in the same amounts.

## 5 Conclusions

In conclusion, the results of this study showed that IND has significant anxiolytic effects with lowering the locomotor movement. Our investigation also demonstrated that the tested compound (IND) exerted anxiolytic effect with dose dependant manner. In addition, during the combination treatment it was clear that IND exhibited synergistic activity on experimental animals. Moreover, according to the *in silico* study the IND displayed the highest binding energy (−7.7 kcal/mol) to bind with the α3 subunit of the GABA_A_ receptor, while DZP showed a lower binding energy (−6.4 kcal/mol). In case of pharmacokinetics parameters, the IND demonstrated a good ADMET profile and successfully meets all the characteristics of the Lipinski rule of five. Furthermore, the toxicity analysis demonstrated that IND shows no toxic effect in cases of immunotoxicity, mutagenicity, cytotoxicity, respiratory toxicity, cardiotoxicity, and ecotoxicity. However, this study suggests doing more experiments to determine the exact mechanisms of action and create a thorough evaluation of the safety and efficacy of this molecule, including determining the optimum dose, before it can be made available as a recommended prescription medicine.

## 6 Funding

Researcher supporting Project (RSPD2024R744), King Saud University, Riyadh, Saudi Arabia.

## Conflict of Interests

Not applicable

7

## Data Availability

Not applicable.

## References

[1] M. Afroz , M. S. Bhuia , M. A. Rahman , R. Hasan , T. Islam , M. R. Islam , R. Chowdhury , M. A. Khan , E. Antas , D. Silva , H. D. Melo Coutinho , M. T. Islam , Eur. J. Pharmacol. 2024, 965, 176289, 10.1016/j.ejphar.2023.176289. 38158111

[2] S. Akash , M. E. Hosen , S. Mahmood , S. J. Supti , A. Kumer , S. Sultana , S. Jannat , I. Bayıl , H. A. Nafidi , Y. A. B. Jardan , A. B. Mekonnen , M. Bourhia , Front. Cell. Infect. Microbiol. 2023, 13, 1222913, 10.3389/fcimb.2023.1222913. 37662005 PMC10469490

[3] T. H. Bak, S. Chandran, *Cortex; a journal devoted to the study of the nervous system and behavior* **2012**, *48 (7)*, 936–944, 10.1016/j.cortex.2011.07.008. 21924711

[4] M. H. Bappi , A. A. S. Prottay , H. Kamli , F. A. Sonia , M. N. Mia , M. S. Akbor , M. M. Hossen , S. Awadallah , M. S. Mubarak , M. T. Islam , Molecules 2023, 28 (14), 5616, 10.3390/molecules28145616. 37513487 PMC10384931

[5] L. M. Behlke , R. A. Foster , J. Liu , D. Benke , R. S. Benham , A. J. Nathanson , B. K. Yee , H. U. Zeilhofer , E. Engin , U. Rudolph , Neuropsychopharmacology: official publication of the American College of Neuropsychopharmacology 2016, 41 (10), 2492–2501, 10.1038/npp.2016.49. 27067130 PMC4987847

[6] C. Belzung , G. Griebel , Behav. Brain Res. 2001, 125 (1–2), 141–149, 10.1016/s0166--4328(01)00291--1. 11682105

[7] M. S. Bhuia , M. Rokonuzzman , M. I. Hossain , S. A. Ansari , I. A. Ansari , T. Islam , M. S. Al Hasan , M. S. Mubarak , M. T. Islam , Pharmaceuticals (Basel) 2023, 16 (9), 1271, 10.3390/ph16091271. 37765079 PMC10535412

[8] M. S. Bhuia , T. Islam , M. Rokonuzzman , A. A. Shamsh Prottay , F. Akter , M. I. Hossain , R. Chowdhury , M. A. Kazi , A. B. R. Khalipha , H. D. M. Coutinho , M. T. Islam , 3 Biotech. 2023, 13 (4), 116, 10.1007/s13205-023-03520-3. PMC10008523 36919029

[9] M. S. Bhuia , M. M. Rahaman , T. Islam , M. H. Bappi , M. I. Sikder , K. N. Hossain , J. Sharifi-Rad , Chin. Med. 2023, 18 (1), 27, 10.1186/s13020-023-00735-7. 36918923 PMC10015939

[10] E. A. Blomme , Y. Will , Chem. Res. Toxicol. 2016, 29 (4), 473–504, 10.1021/acs.chemrestox.5b00407. 26588328

[11] P. Botta , L. Demmou , Y. Kasugai , M. Markovic , C. Xu , J. P. Fadok , T. Lu , M. M. Poe , L. Xu , J. M. Cook , U. Rudolph , P. Sah , F. Ferraguti , A. Lüthi , Nat. Neurosci. 2015, 18 (10), 1493–1500, 10.1038/nn.4102. 26322928 PMC4607767

[12] S. K. Burley , H. M. Berman , G. J. Kleywegt , J. L. Markley , H. Nakamura , S. Velankar , Method. Mol. Biol. (Clifton, N. J.) 2017, 1607, 627–641, 10.1007/978-1-4939-7000-1_26. PMC5823500 28573592

[13] A. Bystritsky , D. Kronemyer , The Psychiatric clinics of North America 2014, 37 (4), 489–518, 10.1016/j.psc.2014.08.002. 25455062

[14] A. Bystritsky , S. S. Khalsa , M. E. Cameron , J. Schiffman , P & T: A Peer-Rev. J. Form. Management 2013, 38 (1), 30–57. PMC3628173 23599668

[15] K. L. Chapman , H. Holzgrefe , L. E. Black , M. Brown , G. Chellman , C. Copeman , J. Couch , S. Creton , S. Gehen , A. Hoberman , L. B. Kinter , S. Madden , C. Mattis , H. A. Stemple , S. Wilson , Regulat. Toxicol. Pharmacol.: RTP 2013, 66 (1), 88–103, 10.1016/j.yrtph.2013.03.001. 23524271

[16] X. Chen , J. van Gerven , A. Cohen , et al., Acta Pharmacol Sin. 2019, 40, 571–582, 10.1038/s41401-018-0185-5. 30518829 PMC6786312

[17] X. Cheng , K. H. Merz , S. Vatter , J. Zeller , S. Muehlbeyer , A. Thommet , J. Christ , S. Wölfl , G. Eisenbrand , J. Med. Chem. 2017, 60 (12), 4949–4962, 10.1021/acs.jmedchem.7b00324. 28557430

[18] E. Choleris , A. W. Thomas , M. Kavaliers , F. S. Prato , Neurosci. Biobehav. Rev. 2001, 25 (3), 235–260, 10.1016/s0149-7634(01)00011-2. 11378179

[19] R. Chowdhury , M. S. Bhuia , A. I. Rakib , R. Hasan , H. D. M. Coutinho , I. M. Araújo , I. R. A. de Menezes , M. T. Islam , Plants (Basel, Switzerland) 2023, 12 (24), 4189, 10.3390/plants12244189. 38140516 PMC10747098

[20] B. H. Clough , S. Zeitouni , U. Krause , C. D. Chaput , L. M. Cross , A. K. Gaharwar , C. A. Gregory , Stem Cells Transl. Med. 2018, 7 (4), 342–353, 10.1002/sctm.17-0229. 29405665 PMC5866944

[21] I. B. da Silva , I. L. Rangel , R. M. de Leite Lima , E. O. Lima , P. L. de Medeiros , S. N. P. Leite , African J. Pharm. Pharmacol. 2016, 10 (11), 200–205, 10.5897/AJPP2015.4386.

[22] A. A. C. de Almeida , J. P. Costa , R. B. F. de Carvalho , D. P. de Sousa , R. M. de Freitas , Brain Res. 2012, 1448, 56–62, 10.1016/j.brainres.2012.01.070. 22364736

[23] J. de Ruyck , G. Brysbaert , R. Blossey , M. F. Lensink , Adv Appl Bioinform Chem 2016, 9, 1–11, 10.2147/AABC.S105289. 27390530 PMC4930227

[24] E. Dean , Anxiety. Nursing standard (Royal College of Nursing (Great Britain): 1987) 2016, 30 (46), 15, 10.7748/ns.30.46.15.s17. 27406490

[25] R. Doron , D. Lotan , A. Rak-Rabl , A. Raskin-Ramot , K. Lavi , M. Rehavi , Life Sci. 2012, 90 (25–26), 995–1000, 10.1016/j.lfs.2012.05.014. 22683433

[26] A. Ebihara , D. Sugihara , M. Matsuyama , C. Suzuki-Nakagawa , A. H. M. N. Nabi , T. Nakagawa , A. Nishiyama , F. Suzuki , Hypertension Res.: Official J. Japan. Soc. Hypertension 2023, 46 (4), 959–971, 10.1038/s41440--022--01094-w. PMC10073018 36481966

[27] A. N. Edinoff , H. A. Akuly , T. A. Hanna , C. O. Ochoa , S. J. Patti , Y. A. Ghaffar , A. D. Kaye , O. Viswanath , I. Urits , A. G. Boyer , et al., Neurol. Int. 2021, 13 (3), 387–401, 10.3390/neurolint13030038. 34449705 PMC8395812

[28] A. Efstathiou , C. S. Meira , N. Gaboriaud-Kolar , T. M. Bastos , V. P. C. Rocha , K. Vougogiannopoulou , M. B. P. Soares , Virulence 2018, 9 (1), 1658–1668, 10.1080/21505594.2018.1532242. 30387370 PMC7000199

[29] J. Fedotova , P. Kubatka , D. Büsselberg , A. G. Shleikin , M. Caprnda , J. Dragasek , P. Kruzliak , Biomed. Pharmacother. 2017, 95, 437–446, 10.1016/j.biopha.2017.08.107. 28863384

[30] N. Gaboriaud-Kolar , K. Vougogiannopoulou , A. L. Skaltsounis , Expert Opin. Ther. Pat. 2015, 25 (5), 583–593, 10.1517/13543776.2015.1019865. 25887337

[31] A. Garakani , J. W. Murrough , R. C. Freire , R. P. Thom , K. Larkin , F. D. Buono , D. V. Iosifescu , Front. Psychiatry 2020, 11, 595584, 10.3389/fpsyt.2020.595584. 33424664 PMC7786299

[32] M. K. Gilson , H. X. Zhou , Annual Rev. Biophys. Biomol. Struct. 2007, 36, 21–42, 10.1146/annurev.biophys.36.040306.132550. 17201676

[33] M. P. Gleeson , A. Hersey , D. Montanari , J. Overington , Nat. Rev. Drug Discovery 2011, 10 (3), 197–208, 10.1038/nrd3367. 21358739 PMC6317702

[34] Z. H. Gong , Y. F. Li , N. Zhao , H. J. Yang , R. B. Su , Z. P. Luo , J. Li , Eur. J. Pharmacol. 2006, 550 (1–3), 112–116, 10.1016/j.ejphar.2006.08.057. 17011547

[35] T. D. Gould, D. T. Dao, C. E. Kovacsics, *The open field test. Mood and anxiety related phenotypes in mice: Characterization using behavioral tests* **2009**, *01*, 1–20. DOI (10.1007/978-1-60761-303-9_1).

[36] M. Gros , B. Gros , J. E. Mesonero , E. Latorre , J. Clin. Med. 2021, 10 (15), 3429, 10.3390/jcm10153429. 34362210 PMC8347293

[37] R. Hasan , A. Alshammari , N. A. Albekairi , M. S. Bhuia , M. Afroz , R. Chowdhury , M. A. Khan , S. A. Ansari , I. A. Ansari , M. S. Mubarak , M. T. Islam , Sci. Rep. 2024, 14 (1), 6642, 10.1038/s41598--024--57173--0. 38503897 PMC10951218

[38] C. Hetényi , M. Bálint , Methods in molecular biology (Clifton, N. J.) 2020, 2112, 107–121, 10.1007/978-1-0716-0270-6_8. 32006281

[39] I. Hoque , A. Chatterjee , S. Bhattacharya , R. Biswas , Int. J. Adv. Res. Biol. Sci. 2017, 4 (2), 60–71.

[40] B. A. Hozack , J. M. Kistler , A. R. Vaccaro , P. K. Beredjiklian , JBJS 2022, 104 (24), 2204–2210, DOI: 10.2106/JBJS.22.00516. 36223476

[41] M. S. Islam , R. Hossain , T. Ahmed , M. M. Rahaman , K. Al-Khafaji , R. A. Khan , M. T. Islam , Molecules 2022, 27 (21), 7149,, 10.3390/molecules27217149. 36363979 PMC9656213

[42] M. T. Islam , R. D. Freitas , G. D. S. Oliveira , B. Guha , J. Pharm. Pharm. Sci. 2014, 3 (3), 62–71.

[43] M. T. Islam , N. Martins , M. Imran , A. Hameed , S. W. Ali , B. Salehi , I. Ahmad , A. Hussain , J. Sharifi-Rad , Cellular and Molecular Biology (Noisy-le-Grand, France) 2020, 66 (4), 73–77. 32583774

[44] G. R. Jang , R. Z. Harris , D. T. Lau , Med. Res. Rev. 2001, 21 (5), 382–396, 10.1002/med.1015. 11579439

[45] Z. Ju , J. Sun , Y. Liu , Molecules 2019, 24 (21), 3831, 10.3390/molecules24213831. 31652913 PMC6865026

[46] B. Karaman , W. Sippl , Eur. J. Med. Chem. 2015, 93, 584–598, 10.1016/j.ejmech.2015.02.045. 25748123

[47] S. Khalifeh , M. S. Pour , A. Ghermezian , A. Behvarmanesh , M. Moghtadaei , G. Ashabi , M. R. Zarrin-Dast , Archives of Adv. Biosci. 2021, 12 (1), 45–51.

[48] M. H. Kim , Y. Y. Choi , G. Yang , I. H. Cho , D. Nam , W. M. Yang , J. Ethnopharmacol. 2013, 145 (1), 214–219, 10.1016/j.jep.2012.10.055. 23149289

[49] J. Knabl , R. Witschi , K. Hösl , H. Reinold , U. B. Zeilhofer , S. Ahmadi , J. Brockhaus , M. Sergejeva , A. Hess , K. Brune , J. M. Fritschy , U. Rudolph , H. Möhler , H. U. Zeilhofer , Nature 2008, 451 (7176), 330–334, 10.1038/nature06493. 18202657

[50] T. Konno , K. Sasaki , K. Kobayashi , T. Murata , Mol. Med. 2020, 21 (3), 1552–1560, 10.3892/mmr.2020.10946. PMC7003043 32016452

[51] O. Korb , T. Stützle , T. E. Exner , J. Chem. Inf. Model. 2011, 51 (4), 865–876, 10.1021/ci100459b. 21434638

[52] A. K. Kraeuter , P. C. Guest , Z. Sarnyai , Pre-Clin. Models: Tech. Protocol. 2019, 1916, 99–103, 10.1007/978-1-4939-8994-2_9. 30535687

[53] A. Krüger , V. Gonçalves Maltarollo , C. Wrenger , T. Kronenberger , Drug Discov. Development-New Adv. 2019, 1, 1–30.

[54] M. Kyrios , R. Mouding , M. Nedeljkovic , Australian Family Physician 2011, 40 (6), 370–374. 21655481

[55] C. A. Lipinski , Drug Discovery Today Technol. 2004, 1 (4), 337–341, 10.1016/j.ddtec.2004.11.007. 24981612

[56] B. Luscher , Q. Shen , N. Sahir , Mol. Psychiatry 2011, 16, 383–406, 10.1038/mp.2010.120. 21079608 PMC3412149

[57] H. Li , H. Zhang , L. Chen , Y. Shen , Y. Cao , X. Li , J. Yao , J. Biomed.’ Res. 2024, 38 (3), 256–268, 10.7555/JBR.37.20230078. 38387889 PMC11144936

[58] L. Magro , M. Faccini , R. Leone , Lormetazepam Addiction. In Neuropathology of Drug Addictions and Substance Misuse, Academic Press, 2016, *3*, 273–282, 10.1016/B978-0-12-800634-4.00027-5.

[59] S. Medina-Moreno , T. C. Dowling , J. C. Zapata , N. M. Le , E. Sausville , J. Bryant , R. R. Redfield , A. Heredia , PLoS One 2017, 12 (8), e0183425, 10.1371/journal.pone.0183425. 28817720 PMC5560554

[60] M. N. Mia , S. Z. Smrity , M. H. Bappi , H. Kamli , T. Islam , A. A. S. Prottay , M. T. Islam , Food Biosci. 2023, 55, 103044, 10.1016/j.fbio.2023.103044.

[61] P. Nuss , Neuropsychiatr. Dis. Treat. 2015, 11, 165–175, 10.2147/NDT.S58841. 25653526 PMC4303399

[62] F. Ohl , Clin. Neurosci. Res. 2003, 3 (4–5), 233–238.

[63] C. N. Patel , S. P. Kumar , R. M. Rawal , D. P. Patel , F. J. Gonzalez , H. A. Pandya , Toxicol. Mech. Methods 2020, 30 (3), 159–166, 10.1080/15376516.2019.1681044. 31618094 PMC7383222

[64] K. Ponnusamy , C. Petchiammal , R. Mohankumar , W. Hopper , J. Ethnopharmacol. 2010, 132 (1), 349–354, 10.1016/j.jep.2010.07.050. 20691774

[65] T. Prueksaritanont , C. Tang , AAPS J. 2012, 14 (3), 410–419, 10.1208/s12248-012-9353-6. 22484625 PMC3385832

[66] L. Prut , C. Belzung , Eur. J. Pharmacol. 2003, 463 (1–3), 3–33, 10.1016/s0014-2999(03)01272-x. 12600700

[67] T. Qi , H. Li , S. Li , Oncotarget 2017, 8 (22), 36658–36663, 10.18632/oncotarget.17560. 28525368 PMC5482685

[68] S. M. Rahman , S. Rana , M. N. Islam , A. Kumer , M. M. Hassan , T. K. Biswas , M. Atikullah , Pharmacol. Pharmacy 2019, 10 (06), 283–297.

[69] A. B. Raies , V. B. Bajic , Wiley Interdiscip. Rev.: Comput. Mol. Sci. 2016, 6 (2), 147–172, 10.1002/wcms.1240. 27066112 PMC4785608

[70] P. W. Reimus , M. A. Dangelmayr , J. T. Clay , K. R. Chamberlain , Environ. Sci. Technol. 2019, 53 (13), 7483–7493, 10.1021/acs.est.9b01572. 31132251

[71] K. J. Ressler , C. B. Nemeroff , Depression Anxiety 2000, 1 (12), 2–19, 10.1002/1520-6394(2000)12:1+2::AID-DA23.0.CO;2-4. 11098410

[72] C. Rockhill , I. Kodish , C. DiBattisto , M. Macias , C. Varley , S. Ryan , Current problems in pediatric and adolescent health care 2010, 40 (4), 66–99, 10.1016/j.cppeds.2010.02.002. 20381781

[73] J. K. Rowlett , D. M. Platt , S. Lelas , J. R. Atack , G. R. Dawson , Proc. Natl. Acad. Sci. USA 2005, 102 (3), 915–920, 10.1073/pnas.0405621102. 15644443 PMC545524

[74] U. Rudolph , F. Crestani , D. Benke , I. Brünig , J. A. Benson , J. M. Fritschy , J. R. Martin , H. Bluethmann , H. Möhler , Nature 1999, 401 (6755), 796–800, 10.1038/44579. 10548105

[75] A. Ruiz-Garcia , M. Bermejo , A. Moss , V. G. Casabo , J. Pharm. Sci. 2008, 97 (2), 654–690, 10.1002/jps.21009. 17630642

[76] V. T. Sabe , T. Ntombela , L. A. Jhamba , G. E. M. Maguire , T. Govender , T. Naicker , H. G. Kruger , Eur. J. Med. Chem. 2021, 224, 113705, 10.1016/j.ejmech.2021.113705. 34303871

[77] E. Sanabria , R. E. Cuenca , M. Á. Esteso , M. Maldonado , Toxics 2021, 9 (2), 25, 10.3390/toxics9020025. 33535485 PMC7912725

[78] M. L. Seibenhener , M. C. Wooten , J. Visualization 2015, (96), e52434, 10.3791/52434. PMC4354627 25742564

[79] S. Sekhon , J. Koo , Br. J. Dermatol. 2018, 178 (1), 21, 10.1111/bjd.16074. 29357584

[80] T. Serchov , D. van Calker , K. Biber , Bio-Protocol 2016, 6 (19), e1957–e1957.

[81] H. Shoji , K. Mizoguchi , Behav. Brain Res. 2010, 211 (2), 169–177, 10.1016/j.bbr.2010.03.025. 20307586

[82] A. Singh , R. Kukreti , L. Saso , S. Kukreti , Molecules 2019, 24, 1583, 10.3390/molecules24081583. 31013638 PMC6514564

[83] O. V. Sobolev , P. V. Afonine , N. W. Moriarty , M. L. Hekkelman , R. P. Joosten , A. Perrakis , P. D. Adams , Structure (London, England: 1993) 2020, 28 (11), 1249–1258.e2, 10.1016/j.str.2020.08.005. 32857966 PMC7642142

[84] V. Sobolev , R. C. Wade , G. Vriend , M. Edelman , Proteins 1996, 25 (1), 120–129, 10.1002/(SICI)1097-0134(199605)25:1120::AID-PROT103.0.CO;2-M. 8727324

[85] N. Subhan , M. A. Alam , F. Ahmed , I. J. Shahid , L. Nahar , S. D. Sarker , Rev. Brasil. Farmacogn. 2008, 18, 521–526.

[86] D. Sun , W. Gao , H. Hu , S. Zhou , Acta Pharm. Sin. B 2022, 12 (7), 3049–3062, 10.1016/j.apsb.2022.02.002. 35865092 PMC9293739

[87] D. M. Teleanu , A. G. Niculescu , I. I. Lungu , C. I. Radu , O. Vladâcenco , E. Roza , B. Costăchescu , A. M. Grumezescu , R. I. Teleanu , Int. J. Mol. Sci. 2022, 23 (11), 5938, 10.3390/ijms23115938. 35682615 PMC9180653

[88] E. M. Toledo , M. Colombres , N. C. Inestrosa , Prog. Neurobiol. 2008, 86 (3), 281–296, 10.1016/j.pneurobio.2008.08.001. 18786602

[89] G. Ulrich-Merzenich , D. Panek , H. Zeitler , H. Vetter , H. Wagner , Indian J. Experimental Biol. 2010, 48 (3), 208–219. 21046973

[90] A. T. Varela , A. M. Simões , J. S. Teodoro , F. V. Duarte , A. P. Gomes , C. M. Palmeira , A. P. Rolo , Mitochondrion 2010, 10 (5), 456–463, 10.1016/j.mito.2010.04.006. 20433952

[91] L. Wang , B. J. Berne , R. A. Friesner , On achieving high accuracy and reliability in the calculation of relative protein-ligand binding affinities. Proceedings of the National Academy of Sciences of the United States of America 2012, 109 (6), 1937–1942, 10.1073/pnas.1114017109. 22308365 PMC3277581

[92] L. Yang , X. Li , W. Huang , X. Rao , Y. Lai , Biomed. Pharmacother. 2022, 151, 113112, 10.1016/j.biopha.2022.113112. 35598366

[93] X. Zhang , Y. Song , Y. Wu , Y. Dong , L. Lai , J. Zhang , B. Lu , F. Dai , L. He , M. Liu , Z. Yi , Int. J. Cancer 2011, 129 (10), 2502–2511, 10.1002/ijc.25909. 21207415

